# Anti-Proliferative and Apoptosis-Inducing Activity of *Acacia Modesta* and *Opuntia Monocantha* Extracts on HeLa Cells

**DOI:** 10.31557/APJCP.2020.21.10.3125

**Published:** 2020-10

**Authors:** Farah Abid, Muhammad Saleem, Christian D. Muller, Mulazim Hussain Asim, Shumaila Arshad, Tahir Maqbool, Faheem Hadi

**Affiliations:** 1 *Department of Pharmacy, Government College University of Faisalabad, Faisalabad, Pakistan. *; 2 *Faculty of Pharmacy, University of Lahore, Defence Road, Lahore, Pakistan. *; 3 *University College of Pharmacy, University of the Punjab, Lahore, Pakistan. *; 4 *Institut Pluridisciplinaire Hubert Curien, UMR 7178 CNRS, Faculté de Pharmacie, Université de Strasbourg, 67401 Illkirch, France. *; 5 *College of Pharmacy, University of Sargodha, Sargodha, Pakistan. *; 6 *Centre of Research in Molecular Medicine, Institute of Molecular Biology & Biotechnology, University of Lahore, Defence Road, Lahore, Pakistan. *

**Keywords:** Anti-cancer effect, anti-oxidant activity, apoptosis

## Abstract

**Background:**

Cancer is one of the leading causes of death in the world. Numerous phytochemicals from plants have shown antineoplastic effects via programmed cell death (apoptosis). The aim of this study was to evaluate the effect of anti-proliferative and apoptosis-inducing activity of *Acacia modesta *and *Opuntia monocantha* against HeLa cells.

**Methods:**

To estimate anti-proliferative activity of the plants against HeLa cells, ethanol solvent was used for the extraction. For the evaluation of anti-proliferative effects, MTT assay was performed with 100, 200, and 400 µg/mL dose. The antioxidant assays including glutathione reductase (GSH), superoxide dismutase (SOD) and catalase were performed. Moreover, enzyme linked immunosorbent assay (ELISA) was performed. Furthermore, immunocytometry P53 and flow cytometry were also carried out to assess the apoptosis in HeLa cell.

**Results:**

MTT assay showed that the groups treated with *Opuntia monocantha* and Acacia modest have less level of toxicity as compared to untreated groups. Antioxidant assays confirmed that GSH, SPD and, catalase activities were quite decreased in treated groups as compared to untreated groups. Similarly, ELISA and apoptosis p53 have shown more pronounced apoptosis effect in treated groups as compared to untreated groups.

**Conclusion:**

Based on above findings, treatment of HeLa cells with these plant extracts induced apoptosis, restricts proliferation, and enhances the anti-oxidative index in post treated cells.

## Introduction

Cancer is an emerging disease with rapid incidence worldwide, characterized by uncontrolled division of cells with low apoptosis activity. Apoptosis is a programmed cell death and induction of apoptosis is quite crucial in cancer as it reduces proliferation of the malignant cells (Bertram, 2000). Significant advancements have been made in the treatment of cancer and many chemotherapeutic agents are available for the cure and control of the cancer. Though these agents are quite useful but they display several side effects like multiple drug resistance and non-specificity (Greenwell and Rahman, 2015). Globally, medicinal plants are extensively used in practice because of their healing effect on various ailments (Barboza et al., 2009). World Health Organization (WHO) has declared that the medicinal plants are the beneficial reserves of important phytochemicals that could have multiple pharmacological activities. Moreover, therapeutic agents obtained from the medicinal plants are relatively safe and economical as compared to the medicines manufactured synthetically. Therefore, there is an urgent need to focus on the drugs prepared from natural plants, as these are inexpensive, harmless, and effective (Arunkumar and Muthuselvam, 2009). In traditional medicine, herbal plants are broadly used in relation to their numerous therapeutic properties, hence increasing the interest of the researchers to explore these plants (Uprety et al., 2010). *Acacia modesta *belongs to the family Mimosaceae, commonly known as Phulahi and locally called Palosa. It is a medium-sized tree that grows on stony grounds and widely found in different parts of India and Punjab, Khyber Pakhtunkhwa (KPK), and Baluchistan provinces of Pakistan (Baquar, 1989). Its various parts are employed for several curative purposes such as cleaning teeth, wounds, venereal diseases, trachoma, oral toothache, leprosy and dysentery (Bukhari et al., 2010; Chopra et al., 1956; Lewis and Elvin-Lewis, 2003; Rahman et al., 1986). *Opuntia monocantha *is a cactus belonging to the Cactaceae family. Several Cactaceae plant parts have been used in traditional Moroccan medicine (Mouhaddach et al., 2018). *Opuntia monocantha *is largely distributed in Paraguay, Argentina, Uruguay, and Brazil while the plants have also been grown in the various sub-tropical and tropical regions for instance China, Cuba, South Africa, Australia and India (Khales and Baaziz, 2005; Valente et al., 2007). Literature showed that this plant exhibited diverse pharmacological activities, such as hyperlipidemia, hypoglycemic, inhibition of stomach ulceration, emollient, and neuroprotective effects through anti-oxidant and anti-inflammatory actions (Cruse, 1973; Frati et al., 1990; Sáenz et al., 2004). In many countries, it has been applied traditionally as the herbal medicine for managing diabetes, burns, bronchial asthma and acid reflux (Kim et al., 2006; Park and Chun, 2001; Yang et al., 2008). 

The present study was aimed at evaluating the potential anticancer effects of the ethanolic extracts of *Acacia modesta *and *Opuntia monocantha *on HeLa Cells lines. The anti-proliferative and possible apoptotic mechanism of these plants was also examined. Our work here could be a source of information for upcoming researches as previously no such properties were documented.

## Materials and Methods

*Preparation of extracts*

In August 2017, *Acacia modesta *and *Opuntia monocantha *plants were collected from botanical garden of Government Collage University of Lahore, Punjab, Pakistan. The plants were identified and authorized by taxonomist Professor Dr. Zaheer-ud-Din, Department of Botany, Government College University Lahore (Voucher no. 3,654 for *Opuntia monocantha *and 3,655 for *Acacia modesta*). The plants were washed with water and dried under shade at room temperature for 20 days in Pharmacology Research Lab-4, University of Lahore, Lahore. After drying, these plants were sieved, pulverized and stored until further use. The dried plants were then soaked, as a whole, in ethanol to obtain the ethanolic extract of the corresponding plants. After maceration of 7 days, the mixture was then filtered through Whatman #1 paper in a flask and tightly capped. Then, these extracts were evaporated at 40-45°C in rotary evaporator and dried at room temperature.

*Treatment*

Treatment was applied in 96 well plates for the evaluation of MTT and antioxidants, and also in 6 well plates for the estimation of apoptosis via flow cytometry at concentration of 100, 200 and 400 µg/mL. All the experiments were repeated thrice.

*Cytotoxicity analysis (MTT assay)*

To calculate cytotoxicity in HeLa cells, 3-(4,5-dimethylthiazol-2-yl)-2,5-diphenyltetrazolium bromide (MTT) assay was performed with different concentrations onto cells cultivated in 96 well plates according to the protocol described before (Maqbool et al., 2019).

*Antioxidant activity*

Antioxidant assays including glutathione reductase (GSH), superoxide dismutase (SOD) and catalase (CAT) assays were analyzed to check oxidative stress after treating cultured cells in 6-well plates. 

*Glutathione reductase (GSH)*

Glutathione reductase (GSH) assay was performed in a 96 well plate with a reaction mixture of 200 μL in each well. A reaction mixture was prepared by mixing the 20 mM KH_2_PO_4_ buffer (pH 7.5), 40 mM EDTA and 10 mM oxidized glutathione. Medium obtained from different experimental groups were added in the reaction mixture. In the end, 20 mM NADPH was added and absorbance was monitored at 340 nm using spectrophotometer and a graph was plotted. 

*Superoxide dismutase (SOD)*

Superoxide dismutase (SOD) activity was also estimated in 96 well plates following as well-established protocol (Maqbool et al., 2019). The reaction mixture was prepared for this assay in which secretome of different experimental groups of post-treatment on HeLa cells were mixed with 100 mM KH_2_PO_4_ buffer at pH 7.8, 0.1 mM EDTA, 13 mM methionine, 2.25 mM nitro-blue tetrazolium chloride (NBT), 60 µM riboflavin. Its Optical density was measured at 560 nm by spectrophotometer.

*Catalase activity*

The activity of catalase was estimated in a 96 well plates that contained 12.5 mM KH_2_PO_4_ at pH 7.0, 31.25 mM H_2_O_2_ and secretome from different experimental groups of HeLa cells were also present. After keeping in light for 45 to 60 seconds, optical density of all experimental groups contained in wells was measured at 240 nm against blank (Shamim and Rehman, 2015).

*Enzyme-linked immunosorbent assay (ELISA)*

Secretome and cellular lysate of post-treated cells were collected and subjected to solid-phase sandwich ELISA. Secretome of HeLa cell line was used for anti-p53 ELISA in a 96-well plate (Corning, USA) by following an established method (Wajid et al., 2015). Briefly, plate was coated with antibody and incubated for 48 h at 4°C and then washed with 1X TBS-T (washing solution) for five min, Then, 200 µL of blocking solution (BSA) was added in each well of the plate and incubated for 30 min. Following incubation, blocking solution was removed and 200 µL of the secretome from different experimental groups was added to each well. After 18 h medium was removed and each well was washed three times and 100 µL of secondary antibody HRP conjugated donkey anti-rabbit secondary antibody (Santa Cruz Biotechnology, USA) was added to each well and the plate was incubated for 12 h at 4°C. After incubation, secondary antibody was removed. After washing 100 µL of chromogenic solution 3,3’,5,5;-tetramethylbenzidine (TMB) (Invitrogen Inc., USA) was added (chromogenic substrate) and incubated for 20 min. In the end, 100 µL of a stop solution of H_2_SO_4_ (0.18 M) was added and absorbance was monitored at 450 nm.

*Immunocytochemistry*

Cells of experimental groups were subjected to immunostaining by the method of Maqbool et al., (2019). The cells were washed with TBS-T three times followed by incubation in 4% para formaldehyde (PFA) for 30 min at room temperature and washed again with TBS-T five times. Then, 5% BSA was added for 25 min to block nonspecific binding. Cells were again washed with TBS-T five times and incubated with anti-VEGF (vascular endothelial growth factor) (Santa Cruz Biotechnology, USA), Primary antibodies for HGF (Abcam; Cambridge, MA), anti-igf antibody (ab40657) abcam, and anti-p53 (Santa Cruz Biotechnology, USA) rabbit polyclonal primary antibodies. The samples were then incubated for 1.5 h at 37°C and washed five times with TBS-T. After an incubation at 37ºC for 1.5 h with FITC Conjugated donkey anti-rabbit secondary antibody (Santa Cruz Biotechnology, USA), cells were washed five times with TBS-T. Cells were stained with 1 µg/mL diamidino-2-phenylindole (DAPI) (Sigma-Aldrich, USA) at room temperature for 15 min then washed five times with TBS-T prior to floid cell imaging station observation (Life Technologies, USA). The nucleus of cell was stained with blue color, cells appeared to be somewhat spindle-like shape in green color.

*Apoptosis detection by capillary cytometry*

HeLa cells (Passage # 09, 0.125 x 10^6^ cells/well) were seeded in well plates and after 24 h were treated with *Opuntia monocantha *and *Acacia modesta *in increasing concentrations of 100, 200, 400 µg/mL. The H_2_O_2_ was added 1 h before cell extraction as positive apoptosis induction control in 3 wells, media removed from each well and collected in respective tubes. Afterwards, each well was washed with 1 mL of PBS and cells were detached by the addition of 500 μL trypsin 1x solution per well. The plates were gently tapped to be detached and 1 mL of media was added. The cells were centrifuged at 1.5 rpm for 5 min and supernatant discarded. Apoptosis induction was assessed by capillary cytometry (Guava EasyCyte Plus 12HT, Guava/Luminex CA, USA). Previously removed supernatants with non-adherent apoptotic cells were returned to detached cells and stained with Annexin IV-FITC (ImmunoTools GmbH, Friesoy the, Germany, Cat No. 31490013) and propidium iodide (PI, Miltenyi Biotec Inc., Auburn, USA, Cat No. 130-093-233) in a volume of 2 µL each. A minimum of 5000 cells was acquired per sample and analyzed with the InCyte software. Apoptosis rates were assessed by capillary cytometry using Annexin V-FITC and PI according to the manufacturer recommendations. Gates were drawn around the appropriate cell population using forward scatter (FSC) versus side scatter (SSC) acquisition dot plot to exclude any debris. To discriminate between negative and positive events in the analysis, a non-stained control sample always accompanied acquisition of the stained cells to define their cut off. Cytometers performances were checked weekly using the Guava easy Check Kit 4500–0025 (Guava/Luminex, Santa Clara, CA, USA). Cells were categorized according to Annexin-V-FITC (green fluorescence) and PI (red fluorescence) labeling on viable (double negative), preapoptotic cells (Annexin V-FITC single-stained cells), necrotic cells (PI single-stained cells), and cells in advanced phases of apoptosis (double-stained cells).

## Results

*Cytotoxicity activity*

**Figure 1.**
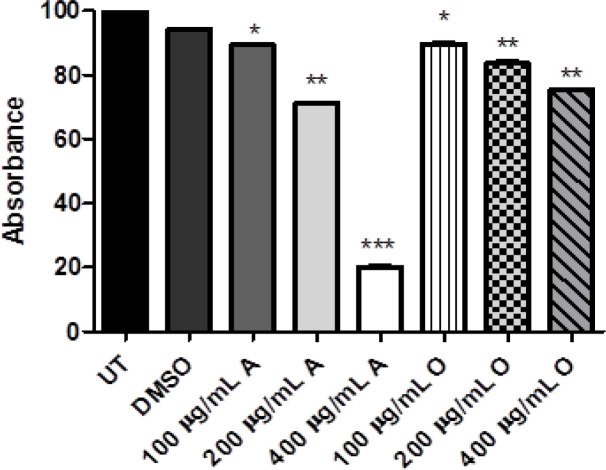
The Cytotoxicity of *Acacia Modesta* (A) and *Opuntia monocantha *(O) plant extracts with treatment ranging from 100-400 µg/mL via MTT assay. According to graph as concentration of plant extract increase the cytotoxicity and can be observed for both plants (A & O), where UT is untreated group and DMSO is treated with dimethyl sulfoxide while "A" represents *Acacia modesta* and "O" *Opuntia monocantha *(p ≤ 0.05)

MTT was performed for evaluating the cytotoxicity via 3-(4, 5-dimethylthiazol-2-yl)-2,5-diphenyltetrazolium bromide. The groups treated with *Opuntia monocantha *and *Acacia modesta *showed reduced viability compared to untreated groups as exhibited in Figure 1. According to the results, extracts showed dose-dependent toxicity as concentration of plant extracts increase more cytotoxicity can be observed in both cases of plants. However, this effect was more pronounced in the case of *Opuntia monocantha *than* Acacia modesta*.

*Antioxidants evaluation*

**Figure 2:**
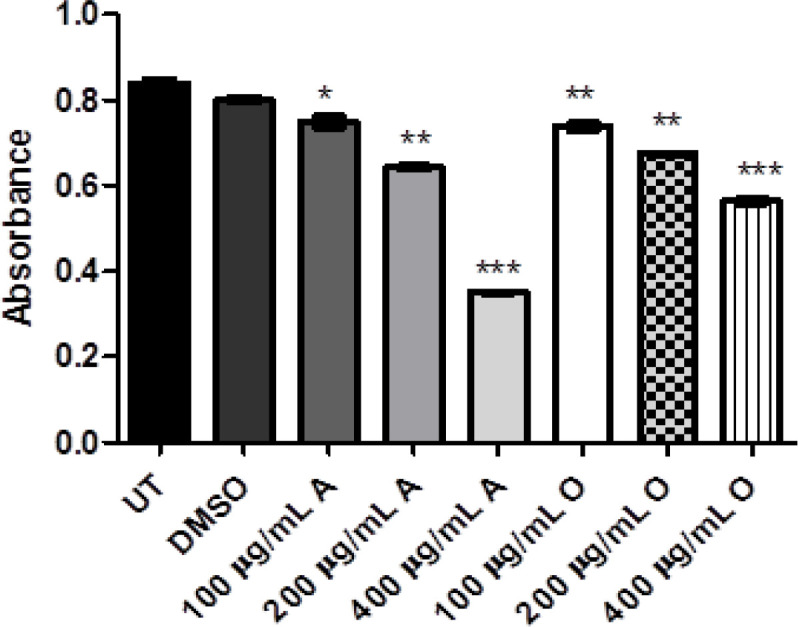
This Figure is Exhibiting Treated HeLa Cells where the Glutathione (Antioxidant) Level of Treated Groups of *Opuntia Monocantha *and *Acacia Modesta* Plant Extracts with Treatment Ranging from 100-400 µg/mL was Observed where Less Glutathione Level was Observed in Treated Groups as Compared with Untreated and DMSO Group. The UT is untreated group and DMSO is treated with dimethyl sulfoxide while "A" represents *Acacia modesta* and "O" *Opuntia monocantha *(p ≤ 0.05)

**Figure 3:**
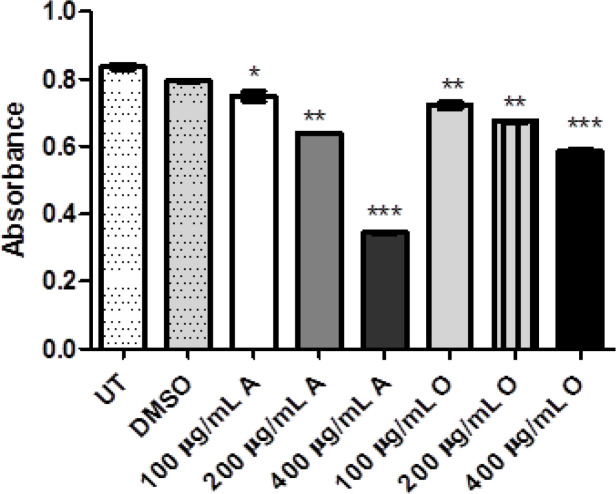
This Figure is Illustrating Treated HeLa Cells where the Superoxide Dismutase (Antioxidant) Level of Treated Groups of *Opuntia Monocantha *and *Acacia Modesta* Plant Extracts with Treatment Ranging from 100-400 µg/mL was Observed where Less Superoxide Dismutase Level was Observed in Treated Groups as Compared with Untreated and DMSO Group. The UT is untreated group and DMSO is treated with dimethyl sulfoxide while "A" represents *Acacia modesta* and "O" *Opuntia monocantha *(p ≤ 0.05)

**Figure 4:**
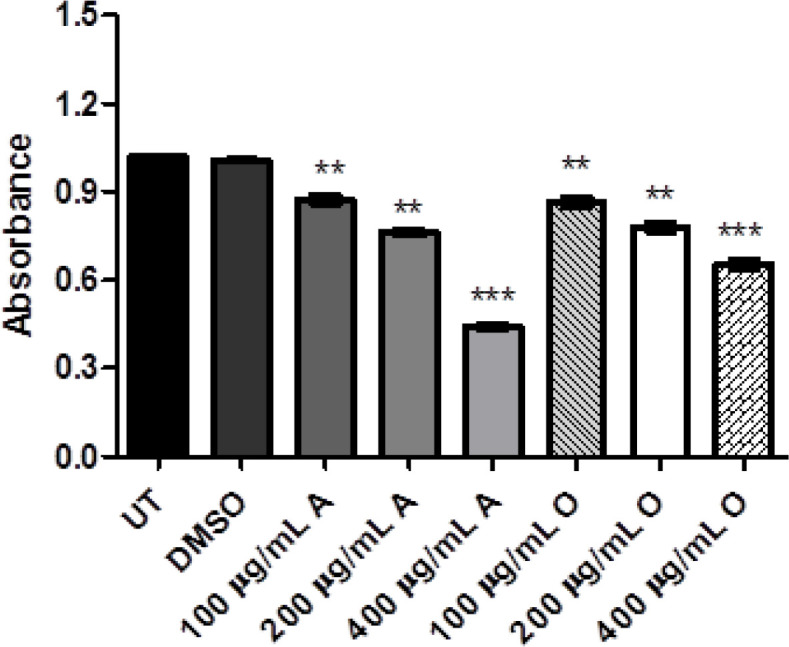
This Figure is Showing Treated Hela Cells where the Catalase (Antioxidant) Level of Treated Groups of *Opuntia Monocantha *and *Acacia Modesta* Plant Extracts with Treatment Ranging from 100-400 µg/Ml was Observed where Less Catalase Level was Observed in Treated Groups as Compared with Untreated and DMSO Group. The UT is untreated group and DMSO is treated with dimethyl sulfoxide while "A" represents *Acacia modesta* and "O" *Opuntia monocantha *(p ≤ 0.05)

The antioxidant potential using the catalase (CAT), superoxide dismutase (SOD) and glutathione reductase (GSH) assays which determine the free radical scavenging activity. It was observed that GSH, CAT, and SOD activities were quite decreased in treated groups as compared to untreated groups as reflected in Figure 2, 3 and 4. Here again, the antioxidant effect produced by both plants was dose-dependent and antioxidant response was more significant via *Acacia modesta *extract as compared to *Opuntia monocantha *extract. Moreover, the antioxidant potential exhibited by catalase, superoxide dismutase and glutathione reductase was almost in the same range.

*Enhanced apoptotic effect of treated HeLa cells via ELISA*

**Figure 5:**
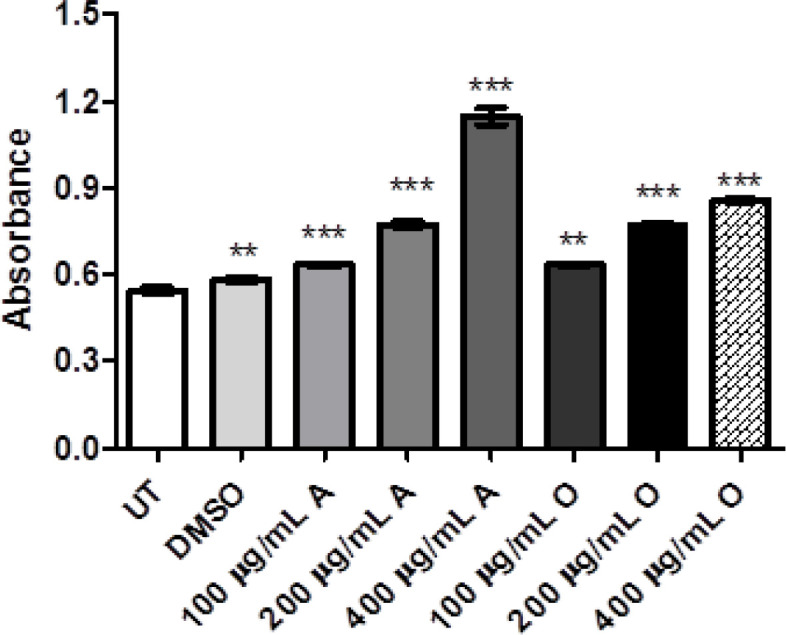
This Figure is Demonstrating Treated HeLa Cells where p53 Level of Treated Groups of *Opuntia Monocantha *and *Acacia Modesta* Plant Extracts with Treatment Ranging from 100-400 µg/mL was Observed where more Release of p53 was Observed in Treated Groups as Compared with Untreated and DMSO Group. The UT is untreated group and DMSO is treated with dimethyl sulfoxide while "A" represents *Acacia modesta* and "O" *Opuntia monocantha *(p ≤ 0.05)

ELISA was performed for evaluating the apoptosis level by the use of p53 antibody, which is a principal factor of apoptosis. Figure 5 showed that treated groups with *Acacia modesta *and *Opuntia monocantha *exhibited higher level of apoptosis as compared to untreated groups and groups treated with DMSO. The apoptotic effect was dose-dependent and the highest apoptosis effect was obtained at dose of 400 µg/mL by *Acacia modesta *extract. Moreover, the effect of *Acacia modesta *was more significant than Opuntia monocantha.

*Enhanced apoptotic effect of treated HeLa cells via immunocytochemistry*

**Figure 6:**
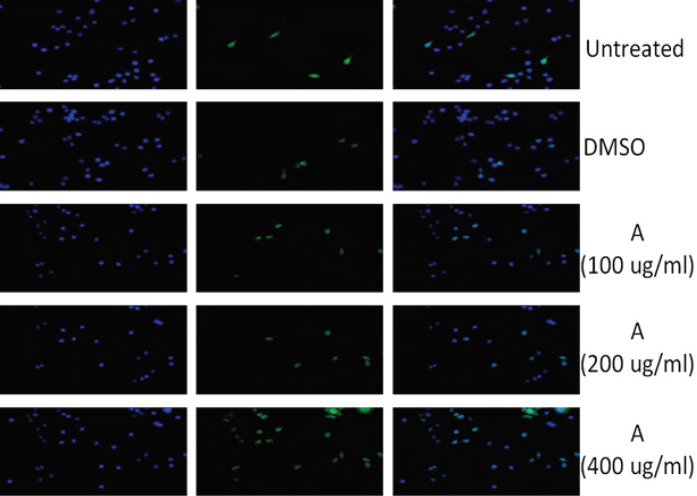
This Figure is Displaying p53 Immunocytochemistry where Blue Color Indicating Nuclear Stain with DAPI Green Color Showing Antibody Stain (p53) and 3^rd^ Group Blue-Green is the Merge of Both DAPI and Antibody Stain. Where 1^st^ group is untreated, 2^nd^ is treated with DMSO, whereas 3^rd^ is treated with *Acacia modesta* (A) at 100 µg/mL dose, 4^th^ group is treated with *Acacia modesta* (A) at 200 µg/mL dose and 5^th^ is treated with *Acacia modesta* (A) at 400 µg/mL dose

**Figure 7:**
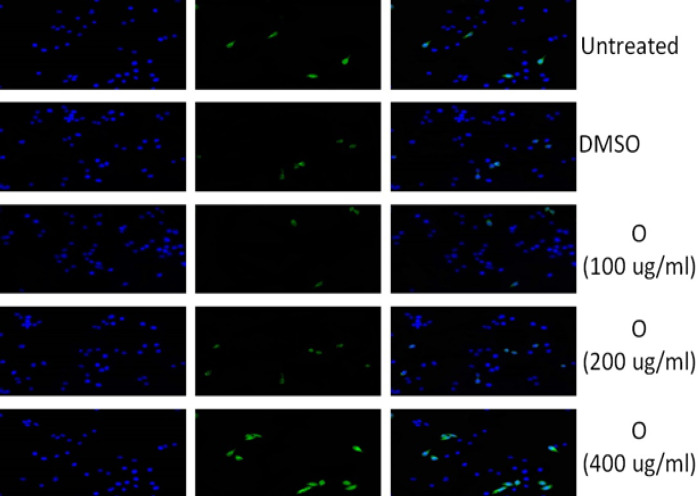
This Figure is Indicating p53 Immunocytochemistry where Blue Color Illustrating Nuclear Stain with DAPI Green Color Showing Antibody Stain (p53) and 3rd Group Blue-Green is the Merge of Both DAPI and Antibody Stain. Where 1^st^ group is untreated 2^nd^ is treated with DMSO 3^rd^ is treated with *Opuntia monocantha *with 100 µg/mL dose 4^th^ is treated with *Opuntia monocantha *with 200 µg/mL doseand 5^th^ is treated with *Opuntia monocantha *with 400 µg/mL dose

Immunocytochemistry was performed for evaluating the apoptosis level by the use of p53 antibody, which is a principal factor of apoptosis. Figure 6 and 7 showed that treated groups with *Acacia modesta *and *Opuntia monocantha *exhibited higher level of apoptosis as compared to untreated groups and groups treated with DMSO. The apoptosis effect was dose-dependent and dose of 400 µg/mL showed higher apoptotic effect.

*Flow cytometry showing enhanced apoptosis effects of HeLa cells*

**Figure 8:**
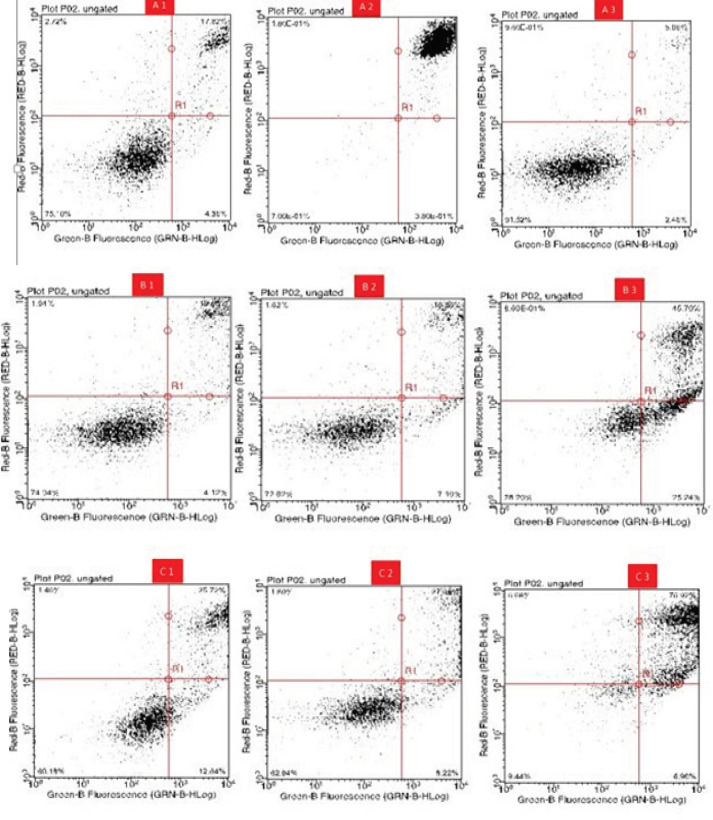
Cytograms Showing Apoptosis Induction in Treated Groups by *Opuntia Monocantha *and *Acacia Modesta *Plant Extracts where A1 is Untreated A2 is H_2_O_2_ and A3 is DMSO, Similarly, B1-B3 is treated with 100, 200 and 400 µg/mL of Opuntia monocantha, respectively and C1-C3 is treated with100, 200 and 400 µg/mL of *Acacia modesta*, respectively

*Acacia modesta *and *Opuntia monocantha *effects were also observed with flow cytometry where more number of dead cells was observed in cells treated with plant extracts compared with untreated and treated with DMSO. Where in case of untreated group there were 75.0% DMSO showing 91.52% *Acacia modesta *with 100 µg/mL dose showing 40% *Acacia modesta *with 200 µg/mL dose showing 32% *Acacia modesta *with 400 µg/mL dose showing 9% and *Opuntia monocantha *with 100 µg/mL dose showing 74% *Opuntia monocantha *with 100 µg/mL dose showing 72% *Opuntia monocantha *with 400 µg/mL dose showing 27% live cells as shown in Figure 8.

## Discussion

One of the leading causes of death worldwide remains cancer. Various therapies have been used including natural products. These natural plants derived compounds provide a new treatments with fewer side effects and even sometimes better efficacy (Wicaksono et al., 2009). New antitumorigenic compounds have extensive effects on proliferation of cancer cells, but it remains very important to address the problematic issue of chemotherapy resistance. Natural compounds from the plants are considered to become the key players in the develolpment of potential drugs for life-threatening diseases. Many cancers in our world are due to dietary imbalance, thus it is very important consideration to discover the new anticancer agent from the natural plant sources that have high antioxidant activities. The main aim of study was to investigate the antiproliferative and apoptotic activity of *Acacia modesta *and *Opuntia monocantha *against HeLa cell line. For this purpose plants were extracted in ethanolic solvent. Our results here indicate that HeLa cells treated with ethanolic plant extracts show significantly reduced level of proliferation. The percentage of dead cells shows that when treated *Acacia modesta *is more efficient as compared to Opuntia monocantha.

Apoptosis induction is a useful strategy for anticancer drug development. Plant-derived anticancer drugs exert cell death by inducing apoptosis in cancer cells. Many mechanisms responsible for apoptosis induced by plants and most of them induce apoptotic cell death by intrinsic or extrinsic pathway and p53 dependent or independent pathway. We also used Annexin V staining for apoptosis determination, in our finding it was observed that plant extracts of *Acacia modesta *and *Opuntia monocantha *induced apoptosis in HeLa cells via p53 pathway. In our study we also did the antioxidant evaluation of plants by SOD, catalase and GSH, as oxidative stress is a primary marker for cancer (Bailey et al., 2012; Lendahl et al., 2009; Toyokuni, 2008). Anti-oxidative enzymes effect the proliferation of cells positively. But when anti-oxidants are given with anti-proliferative therapy it will enhance the effectiveness of therapy and improve anticancer effect. The SOD, catalase and GSH activities were increased when treated with plants *Acacia modesta *and *Opuntia monocantha *extracts. 

Thus our study shows that *Acacia modesta *and *Opuntia monocantha *ethanolic extract exhibit anti-proliferative and pro-apoptotic activity with augmentation of antioxidant activity. Moreover, *Acacia modesta *shows more efficacies as compared to Opuntia monocantha. 

In conclusion, In the present study anti-cancer activity of ethanolic extracts of *Acacia modesta *and *Opuntia monocantha *on HeLa Cells lines have been investigated indicating that these plant extracts are promising in reducing cancer cells growth. According to our findings, treatment of HeLa cells with these plants induces apoptosis, restricts proliferation, and enhances the anti-oxidative index in post-treated cells.
